# Case Report: Life-threatening neutropenia with absolute neutrophil count of zero after low-dose regional arterial infusion chemotherapy to lung in a patient with advanced triple-negative breast cancer after multiple lines of treatment

**DOI:** 10.3389/fonc.2026.1872193

**Published:** 2026-09-11

**Authors:** Yiran Wang, Qianlin Liu, Yanjun Feng, Jun Zhou

**Affiliations:** Department of Gastrointestinal Oncology, Shanghai Gobroad Cancer Hospital, Shanghai, China

**Keywords:** case report, granulocyte colony-stimulating factor, metastatic breast cancer, myelosuppression, regional arterial infusion chemotherapy

## Abstract

**Background:**

The risk of severe myelosuppression following regional arterial perfusion chemotherapy in heavily pre-treated patients is often underestimated, especially when pre-treatment hematologic status appears adequate. This case highlights the unexpectedly profound bone marrow toxicity that can occur under these circumstances and details the critical management strategies required.

**Case presentation:**

We report a case of a 51-year-old female patient with advanced triple negative breast cancer (TNBC) who developed severe myelosuppression following regional arterial perfusion chemotherapy, despite the chemotherapy dose being lower than the standard systemic dose. The patient had grade I myelosuppression before regional arterial perfusion chemotherapy; however, after perfusion with albumin paclitaxel and gemcitabine, she developed life-threatening severe myelosuppression, with absolute neutrophil count (ANC) dropping to 0 and remaining ≤0.01×10^9^/L for 3 consecutive days. Timely administration of granulocyte colony-stimulating factor (G-CSF), antibiotics, and supportive care effectively reversed the myelosuppression and prevented infection. Due to chest tightness and dyspnea caused by the massive pleural effusion, thoracentesis drainage was decisively performed when the white blood cell count showed an upward trend (neutrophil count, 0.06×10^9^/L). The procedure was smooth and without complications.

**Conclusion:**

This case underscores that locoregional chemotherapy, even at reduced doses, can cause profound myelosuppression in extensively pre-treated patients. It also illustrates that a pre-treatment ANC within the normal range may not reliably reflect bone marrow reserve in this population. Prompt, intensive supportive care—including prophylactic antibiotics, G-CSF, and protective isolation—is essential. In carefully selected patients during early hematologic recovery, under strict infection prevention measures and multidisciplinary evaluation, invasive procedures such as thoracentesis may be feasible.

## 1 Introduction

Triple-negative breast cancer accounting for 15–20% of all breast cancers, is characterized by the absence of estrogen receptor (ER), progesterone receptor (PR), and human epidermal growth factor receptor 2 (HER2) expression, leading to limited targeted therapy options and poor prognosis (1). For advanced TNBC, systemic chemotherapy remains the mainstay of treatment (2), but the efficacy decreases significantly after multiple lines of therapy, and treatment-related adverse events become more severe, including myelosuppression (3). Regional arterial perfusion chemotherapy is a minimally invasive treatment that delivers high concentrations of chemotherapy drugs directly to tumor lesions through local arteries, improving the local drug concentration while reducing systemic toxicity (3). In metastatic breast cancer, regional arterial infusion is primarily used for selected patients with liver-dominant or lung-dominant metastases who have progressed after systemic therapy, though its role remains palliative and investigational (4). It is considered to have fewer systemic adverse reactions compared with systemic chemotherapy (5).

Myelosuppression is one of the most common and potentially life-threatening adverse reactions of chemotherapy, which can increase the risk of infection, bleeding, and treatment interruption (6). However, severe myelosuppression induced by regional arterial perfusion chemotherapy with substandard systemic doses in heavily pretreated TNBC patients has rarely been reported. Moreover, the management of persistent neutropenia (ANC ≤0.01×10^9^/L) and the safety of invasive procedures during myelosuppression recovery remain clinical challenges.

Here, we report a case of advanced TNBC patient who developed severe myelosuppression after regional arterial perfusion chemotherapy, and summarize the clinical experience and lessons learned, aiming to provide reference for the clinical management of similar cases. To our knowledge, reports of life-threatening agranulocytosis following pulmonary regional arterial infusion chemotherapy in heavily pre-treated TNBC patients remain extremely limited.

## 2 Case description

### 2.1 Summary of treatment history

**Figure 1:**
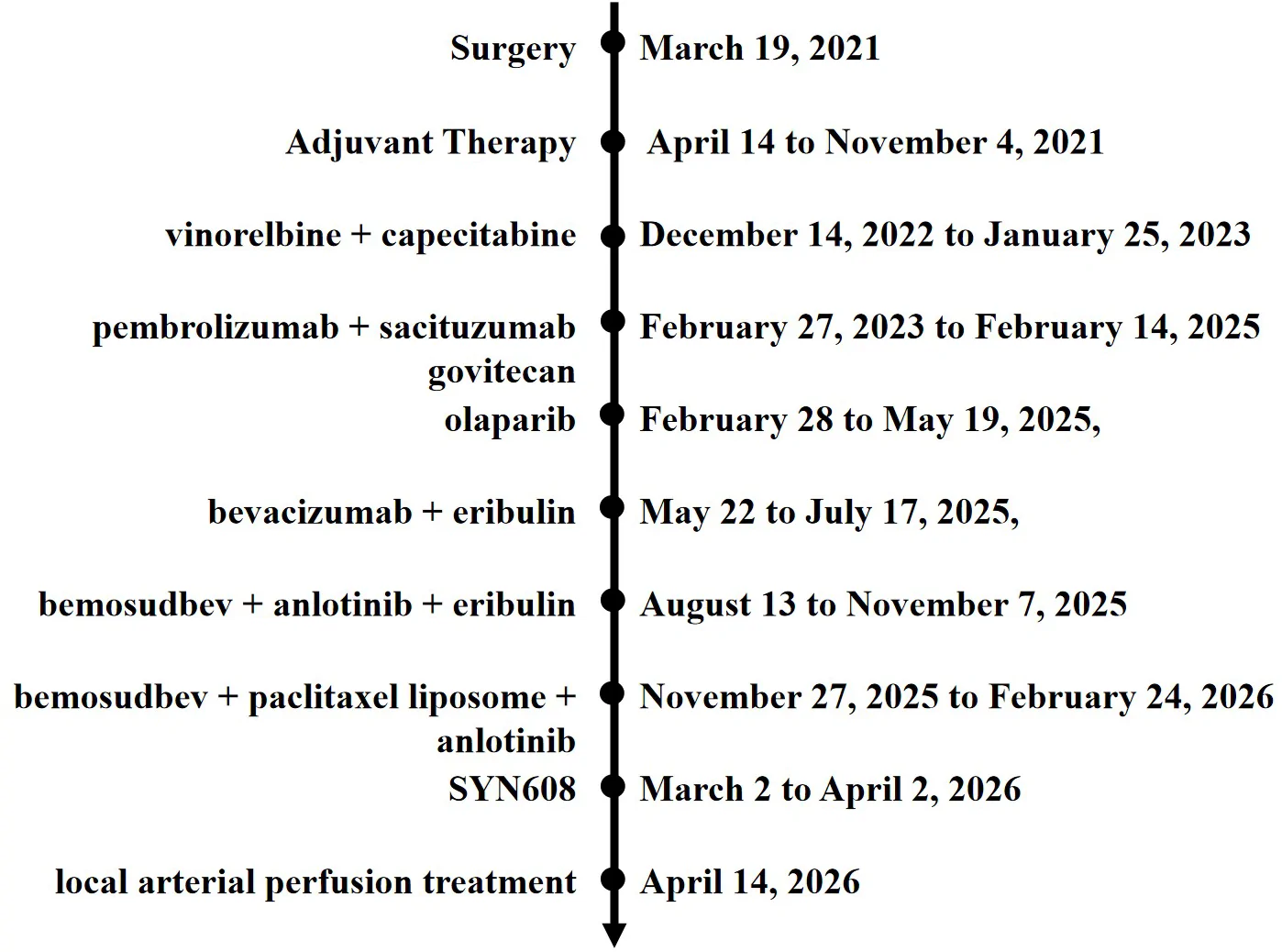
Patient’s treatment regimen since disease onset (March 2021 – August 2026). The timeline illustrates the sequential lines of systemic therapy, radiotherapy, and surgical interventions received by the patient over the course of her disease, including anthracycline-based chemotherapy, taxane/platinum therapy, targeted therapy (sacituzumab govitecan, olaparib, bevacizumab, eribulin, anlotinib, bemosudbev), and investigational agents. RAIC, regional arterial infusion chemotherapy (performed on April 10, 2026).

A female patient, born in 1975, was diagnosed with TNBC in March 2021. Her height has been 162 cm, and her weight has remained stable between 72 and 75 kg over the past five years. She had no history of other chronic diseases or family history of malignant tumors. The patient provided written informed consent for the publication of this case report and all related clinical data. Key chronological treatment events are summarized below (detailed timeline shown in **Figure 1**):

- March 2021: Diagnosis of TNBC; underwent breast-conserving surgery + sentinel lymph node biopsy. Postoperative pathology showed invasive ductal carcinoma (non-special type), grade III, size 4×3×2.8cm, vascular invasion (+), surgical margin (-). IHC: ER (-), PR (-), HER2 (0), Ki-67 (~70%+). Axillary lymph node metastasis: 0/3. Genetic testing: TP53 mutation, no BRCA1/2 exon amplification or deletion.
- April–September 2021: Received 4 cycles of epirubicin + cyclophosphamide, followed by 4 cycles of docetaxel + carboplatin.
- October–November 2021: Received whole-breast radiotherapy (15 fractions) and tumor bed radiotherapy (4 fractions).
- December 2022 – January 2023: Received 4 cycles of vinorelbine + capecitabine.
- February 2023 – February 2025: Received 32 cycles of pembrolizumab + sacituzumab govitecan (dose reduced from 540mg to 360mg from the 5th cycle due to grade II myelosuppression); disease remained stable during this period.
- February–May 2025: Olaparib treatment initiated after genetic testing revealed BRCA1 p.N354Vfs.21 germline mutation, TP53 p.R110Pfs*14, NF1, PBRM1 mutations; MSS; TMB 1.82.
- May–August 2025: Received bevacizumab + eribulin, followed by superficial tumor resection of the left upper arm (pathology confirmed metastatic carcinoma, IHC: PR-, ER-, HER2 0).
- August 2025 – February 2026: Received bemosudbev + anlotinib + eribulin, with paclitaxel liposome added in November 2025; disease progression documented on February 24, 2026.

February 27, 2026: Whole-body PET/CT revealed extensive metastases including bilateral pulmonary nodules, pleural and diaphragmatic metastases, lymph node metastases (right neck, mediastinum, bilateral hila, right cardiophrenic angle, left axilla), sternal soft tissue metastasis, right hepatic lesion, and left upper arm subcutaneous nodule. Also noted chronic inflammatory changes, right pleural effusion, and right lower lobe atelectasis.

- March–April 2026: Enrolled in phase I clinical trial of SYN608; discontinued on April 2, 2026 due to disease progression and massive pleural effusion causing dyspnea. Oxygen saturation around 90%; ultrasound showed right pleural effusion (deepest 116mm) and left pleural effusion (deepest 65mm). Right thoracentesis and catheterization performed on April 2 but dyspnea remained severe.

### 2.2 Decision for regional arterial infusion

**Figure 2:**
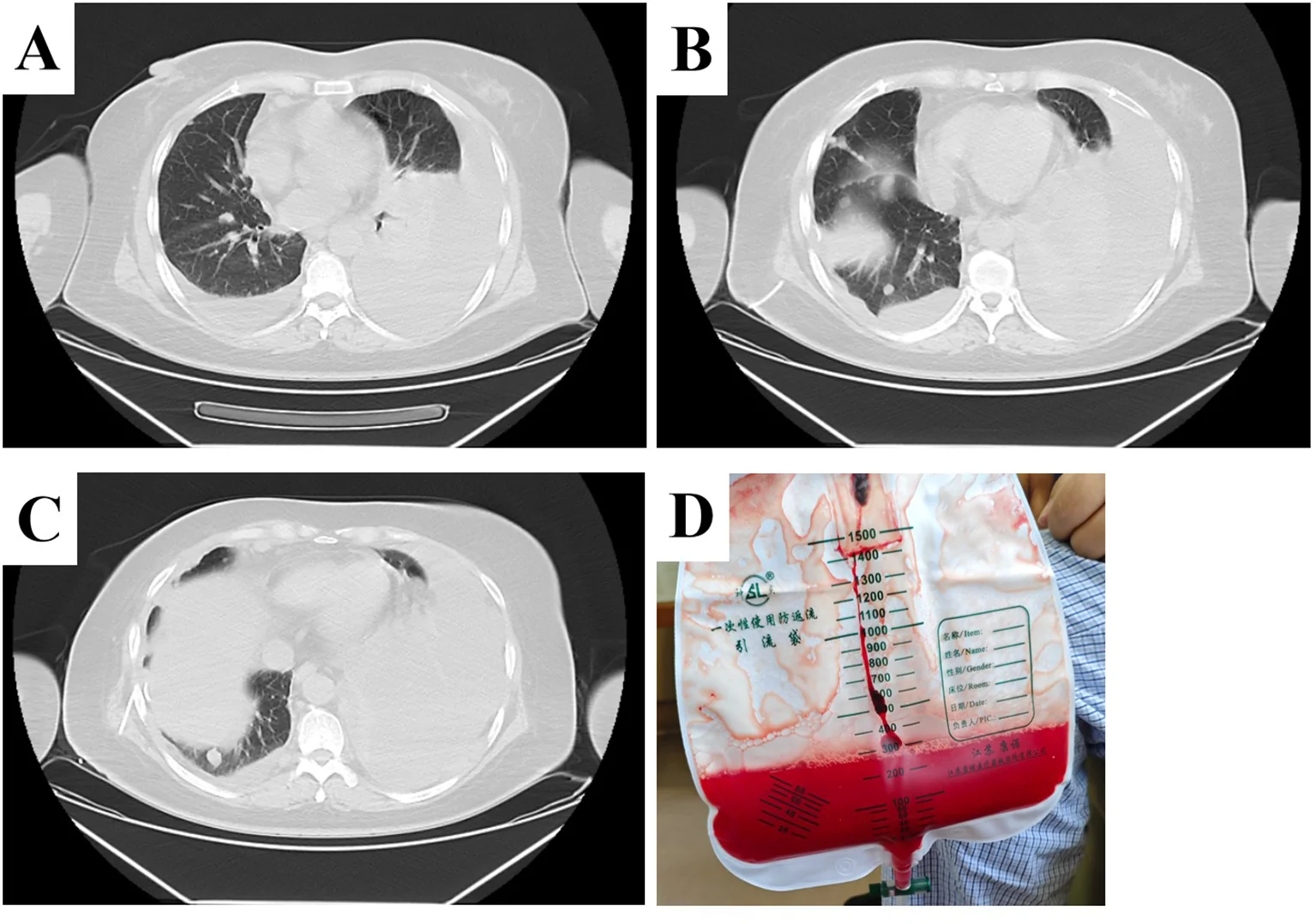
Pre-arterial infusion CT images (April 2026) and drained pleural effusion. **(A–C)** Chest CT images demonstrating extensive bilateral pulmonary metastatic nodules, pleural thickening, and large right-sided pleural effusion with associated right lower lobe atelectasis prior to regional arterial infusion chemotherapy. **(D)** Photograph of the hemorrhagic pleural effusion drained from the right thoracic cavity, consistent with malignant etiology.

Considering the patient had received multiple lines of treatment with unsatisfactory response to intravenous therapy, and presented with rapidly progressing pulmonary lesions and massive bloody pleural effusion (**Figure 2**), the multidisciplinary team (MDT) recommended regional arterial infusion therapy to the lung.

### 2.3 Diagnostic assessment

The diagnosis of metastatic TNBC with pulmonary and pleural progression was established based on the following: (a) histopathological confirmation from multiple biopsies (breast primary in 2021, left arm metastasis in 2025), all consistently showing ER−/PR−/HER2− phenotype; (b) serial imaging (CT and PET/CT) demonstrating progressive pulmonary nodules, pleural thickening, and increasing pleural effusion; and (c) clinical correlation with the patient’s deteriorating respiratory status.

The diagnostic challenges in this case included: (1) distinguishing malignant pleural effusion from paramalignant effusion—the hemorrhagic nature of the drained fluid and the presence of malignant cells on cytology supported a malignant etiology; (2) assessing whether the rapidly progressive dyspnea was primarily due to pulmonary metastases versus massive pleural effusion versus a combination of both—the MDT consensus was that both contributed significantly; and (3) evaluating bone marrow reserve in a heavily pretreated patient—while peripheral blood counts provided a superficial assessment, no bone marrow biopsy was performed to directly evaluate hematopoietic reserve, representing a limitation.

Differential diagnoses considered for the patient’s presentation included: disease progression of TNBC (most likely); treatment-related pulmonary toxicity (less likely given the pattern of nodular progression and pleural involvement); and infectious etiologies (ruled out by absence of fever, negative cultures, and lack of response to antibiotics alone). The prognosis at the time of RAIC was poor, with an expected survival of weeks to months given the rapidly progressive disease after multiple lines of therapy.

### 2.4 Peri-infusion hematologic events

**Figure 3:**
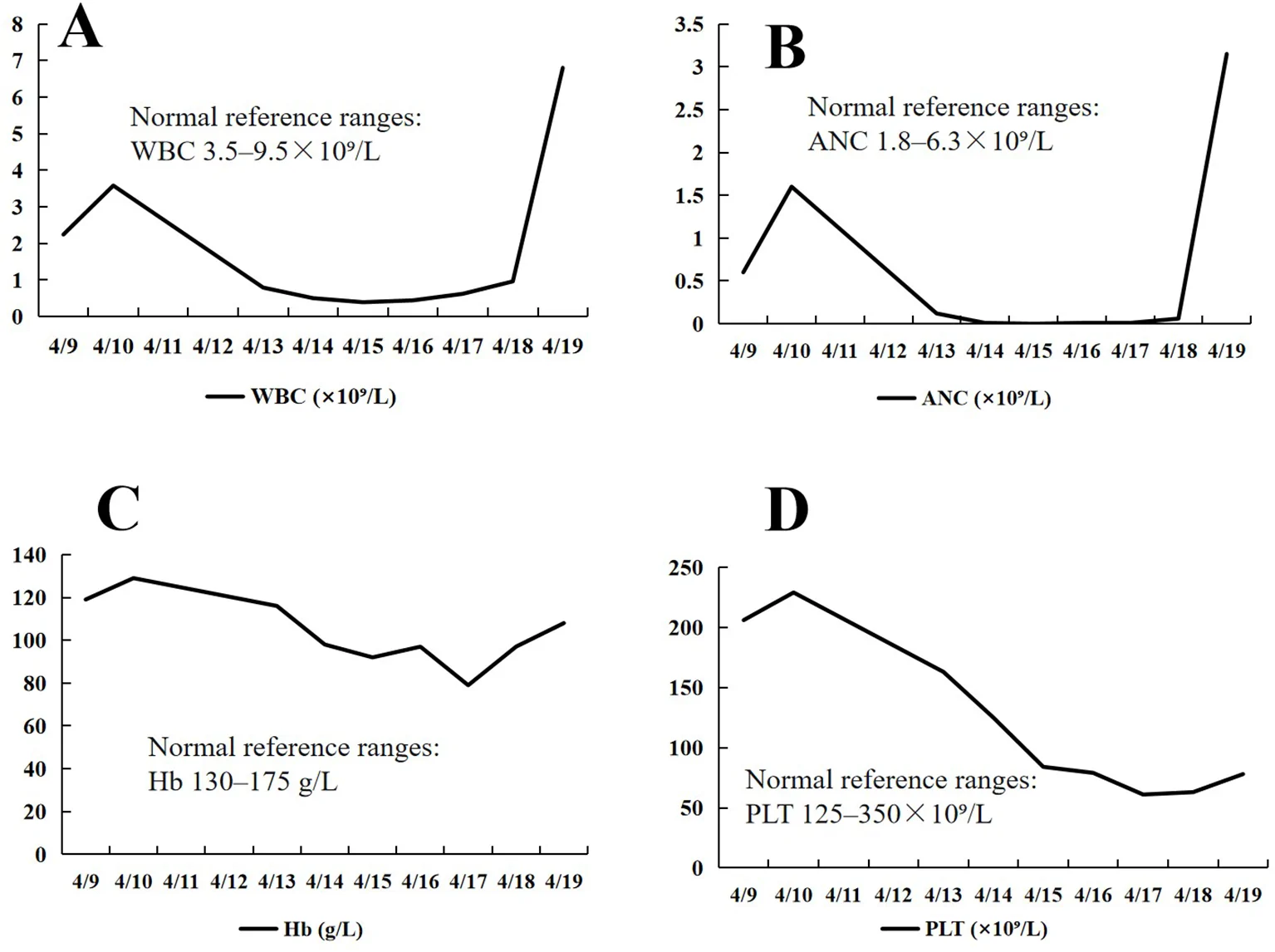
Changes in hematologic parameters after arterial infusion (April 9–19, 2026). **(A)** White blood cell (WBC) count; **(B)** Absolute neutrophil count (ANC); **(C)** Hemoglobin (Hb); **(D)** Platelet (PLT) count. Horizontal dashed lines indicate CTCAE grade thresholds: grade 3 neutropenia (ANC <1.0×10^9^/L), grade 4 neutropenia (ANC <0.5×10^9^/L). Normal reference ranges: WBC 3.5–9.5×10^9^/L; ANC 1.8–6.3×10^9^/L; Hb 130–175 g/L; PLT 125–350×10^9^/L. The shaded area indicates the period of maximal supportive care (April 14–18). RAIC, regional arterial infusion chemotherapy (performed on April 10). G-CSF, granulocyte colony-stimulating factor.

On April 9, 2026, WBC was 2.24×10^9^/L, ANC was 0.6×10^9^/L (grade III myelosuppression). On the same day, hemoglobin was 122 g/L and platelet count was 206 ×10^9^/L. G-CSF 300μg was administered. On April 10, WBC rose to 3.58×10^9^/L and ANC to 1.6×10^9^/L. On the same day, following MDT discussion, regional arterial perfusion chemotherapy of the pulmonary artery was performed with albumin paclitaxel 100mg and gemcitabine 1g (doses lower than standard systemic doses: 226mg and 1.8g respectively for this patient’s BSA of 1.81 m²). The decision was based on the following rationale: (1) the patient’s respiratory status was rapidly deteriorating due to massive malignant effusion and pulmonary metastases, with no effective systemic therapy available at that time; (2) postponing treatment was deemed potentially detrimental given the pace of clinical decline; (3) alternative locally effective interventions were not feasible; and (4) the expected benefit of rapid disease control was considered to outweigh the hematologic risk, with intensive supportive care planned in advance. This decision was reviewed and approved by the institutional MDT. Subsequently, the patient developed severe myelosuppression, and the changes of WBC, ANC, hemoglobin (Hb) and platelets (PLT) are shown in **Figure 3**. On April 13, three days post-procedure, routine blood tests revealed a dramatic decline, with a WBC of 0.79 × 10^9^/L and an ANC of 0.12 × 10^9^/L, signifying Grade 4 neutropenia. A subcutaneous injection of 300 µg granulocyte colony-stimulating factor (G-CSF) and prophylactic intravenous cefoperazone-sulbactam were immediately initiated. Despite this, the cell counts continued to fall.

### 2.5 Sequential changes in therapeutic interventions

Therapeutic interventions were escalated in a stepwise manner based on the severity of myelosuppression (detailed timeline shown in **Table 1**):

**Table 1:** Timeline of the index care episode (April 2–19, 2026).

Date	Event	Key laboratory data	Intervention
Apr 2	Right thoracentesis and catheterization; trial discontinuation	SpO_2_ ~90%; pleural effusion: right 116mm, left 65mm	Catheter drainage
Apr 9	Grade 3 neutropenia detected	WBC 2.24×10^9^/L; ANC 0.6×10^9^/L	G-CSF 300μg SC
Apr 10	Hematologic improvement; RAIC performed	WBC 3.58×10^9^/L; ANC 1.6×10^9^/L	Albumin paclitaxel 100mg + gemcitabine 1g (IA)
Apr 13	Grade 4 neutropenia	WBC 0.79×10^9^/L; ANC 0.12×10^9^/L	G-CSF 300μg SC; cefoperazone-sulbactam IV
Apr 14	Nadir; maximal supportive care activated	ANC 0.01×10^9^/L	Laminar airflow; long-acting G-CSF + split-dose short-acting G-CSF; IVIG; meropenem + caspofungin
Apr 15	ANC 0 (Day 1)	ANC 0×10^9^/L	Romiplostim 250μg (for PLT 84×10^9^/L)
Apr 16	ANC 0 (Day 2)	ANC 0×10^9^/L	Continued maximal support
Apr 17	ANC 0 (Day 3)	ANC 0×10^9^/L	Erythropoietin 5000U started
Apr 18	Early recovery; left thoracentesis performed	WBC 0.96×10^9^/L; ANC 0.06×10^9^/L	Therapeutic thoracentesis (uneventful)
Apr 19	Hematologic recovery	WBC 6.8×10^9^/L; ANC 3.15×10^9^/L	G-CSF and antibiotics discontinued

Level 1 (April 13, upon ANC 0.12×10^9^/L): Short-acting G-CSF 300μg SC + prophylactic cefoperazone-sulbactam IV.

Level 2 (April 14, upon ANC 0.01×10^9^/L): Escalation to combination G-CSF (long-acting Efbemalenograstim alfa 20mg + split-dose short-acting G-CSF 450μg IV in the morning and 150μg SC in the evening) + IVIG 20g daily for passive immunity + immediate transfer to laminar airflow ward. Antibiotics escalated from cefoperazone-sulbactam to meropenem 1g q8h; caspofungin 50mg daily added for antifungal coverage.

Level 3 (April 15, upon PLT 84×10^9^/L [grade 3 thrombocytopenia]): Romiplostim 250μg SC added; erythropoietin 5000U SC started on April 17 for concurrent anemia.

De-escalation (April 19, upon ANC 3.15×10^9^/L): G-CSF and antibiotics discontinued; patient discharged from laminar airflow unit.

The patient’s ANC remained at 0 for three consecutive days (April 15–17). Throughout this period of agranulocytosis, the patient remained afebrile and did not develop any documented infection. On April 18, while still profoundly neutropenic (ANC 0.06×10^9^/L), the patient developed severe respiratory distress from bilateral pleural effusions, with oxygen saturation dropping to 90%. With maximal supportive care in place and the first signs of a rising ANC, a guarded decision was made to perform therapeutic left-sided thoracentesis. The procedure was executed without complication. On April 19, WBC rose to 6.8×10^9^/L and ANC to 3.15×10^9^/L, indicating a robust response to growth factors (**Figure 3**). No other sites of infection were identified on clinical examination or imaging.

**Figure 4:**
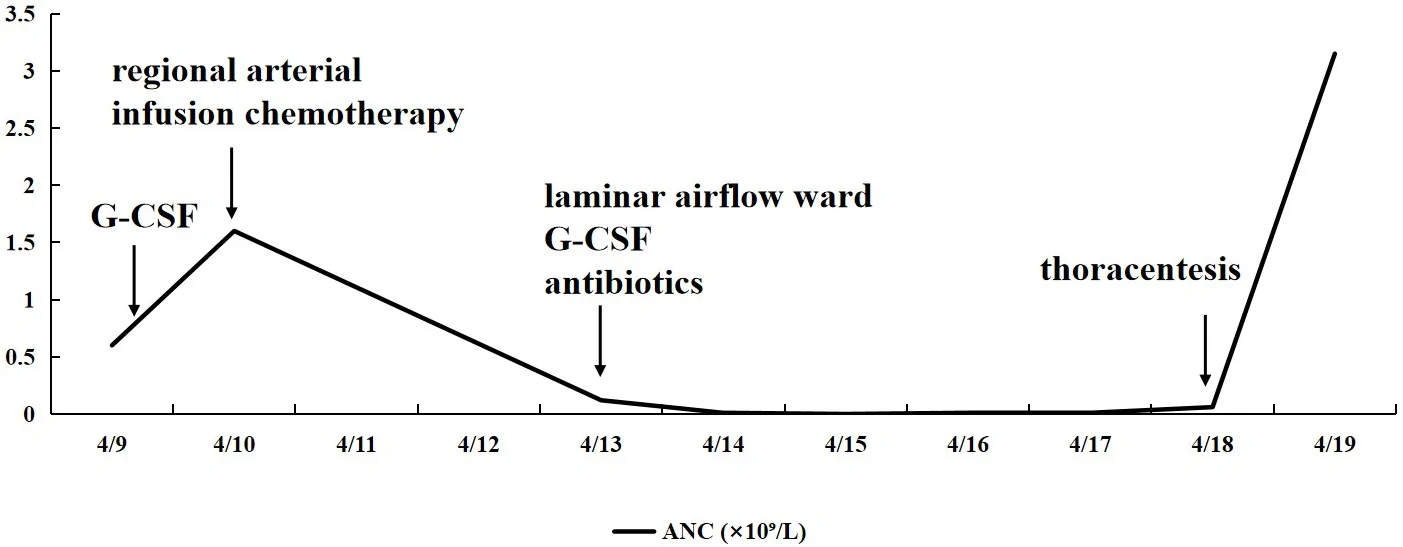
Therapeutic measures before and after arterial infusion. Timeline of sequential interventions during the index care episode, including: (1) pre-infusion G-CSF administration (April 9); (2) RAIC with albumin paclitaxel and gemcitabine (April 10); (3) stepwise escalation of supportive care: Level 1 (April 13, short-acting G-CSF + cefoperazone-sulbactam); Level 2 (April 14, combination G-CSF + IVIG + laminar airflow + meropenem + caspofungin); Level 3 (April 15, romiplostim; April 17, erythropoietin); and (4) de-escalation and discharge from laminar airflow unit upon hematologic recovery (April 19). G-CSF, granulocyte colony-stimulating factor; IVIG, intravenous immunoglobulin; RAIC, regional arterial infusion chemotherapy.

A clear hematologic recovery was observed on April 19, with the WBC surging to 6.8 × 10^9^/L and ANC to 3.15 × 10^9^/L, indicating a robust response to growth factors (**Figures 3**, **4**). The G-CSF and antibiotics were stopped, and the patient’s clinical condition, including dyspnea, improved markedly. She was successfully discharged from the laminar flow unit and remained infection-free. The chest drain was removed, and breathing stabilized. This episode demonstrated that a patient-centric, highly intensive, and proactive supportive strategy could forestall fatal infection during the most extreme period of vulnerability. After recovery from myelosuppression, the patient resumed systemic therapy with Izalontamab Brengitecan (EGFR×HER3 bispecific ADC) on 23^rd^ May. At the time of last follow-up (August 20th, 2026), the patient’s ECOG PS score was 1.

## 3 Discussion

Myelosuppression is a common adverse reaction of chemotherapy, and its severity is related to the type, dose, and administration route of chemotherapy drugs, as well as the patient’s bone marrow reserve function (7). For advanced TNBC patients who have undergone multiple lines of treatment, bone marrow reserve function is often impaired, and the risk of myelosuppression is significantly increased (8). Regional arterial perfusion chemotherapy is a local treatment method that can improve the local drug concentration and reduce systemic toxicity, and is widely used in the treatment of advanced solid tumors (9, 10). Previous studies have shown that regional arterial perfusion chemotherapy has fewer systemic adverse reactions than systemic chemotherapy, and myelosuppression is mostly mild to moderate (11). However, in this case, severe myelosuppression occurred after regional arterial perfusion chemotherapy with substandard systemic dose, which is inconsistent with previous reports. The key lessons from this case are fourfold.

First, this case highlights the potential myelotoxic risk of regional therapy in heavily pre-treated patients. For this patient, with a BSA of 1.81 m², standard intravenous doses would have been 226mg of nab-paclitaxel and 1.8g of gemcitabine; only 100mg and 1g were administered, yet profound myelosuppression (ANC 0) occurred. While regional delivery is thought to reduce systemic exposure, systemic absorption from the pulmonary circulation still occurs. The severity of toxicity in this case is likely attributable to the cumulative effect of over four years of almost continuous cytotoxic and targeted therapy—including anthracyclines, platinums, sacituzumab govitecan, olaparib (a PARP inhibitor), eribulin, and an investigational agent—which had already impaired bone marrow reserve. It is important to acknowledge that cumulative bone marrow exhaustion is an equally plausible explanation for the observed myelosuppression, and causality cannot be definitively attributed to regional arterial infusion alone in a single case. The combination of reduced marrow reserve and any additional genotoxic stress, whether from regional or systemic chemotherapy, may be sufficient to precipitate severe toxicity. While the temporal relationship between RAIC and the development of ANC 0 is suggestive, the contribution of cumulative prior therapy cannot be quantified. In a single case, causality cannot be definitively established; rather, the observation should be interpreted as the combined effect of multiple contributing factors, with RAIC serving as the proximate trigger in a setting of severely depleted marrow reserve.

Second, this case illustrates a potential discrepancy between peripheral ANC and bone marrow reserve. The patient met the conventional ANC threshold of 1.5×10^9^/L for chemotherapy administration, yet experienced a catastrophic nadir (12, 13). This observation suggests that in heavily pre-treated patients, a “normal-range” ANC may represent a stressed, compensatory state rather than true functional reserve. The rapid decline from 1.6×10^9^/L to 0 within days may be a more relevant warning sign than the absolute pre-treatment value. However, as this is a single observation, it cannot challenge current international guidelines; rather, it emphasizes the need for heightened vigilance when administering further chemotherapy to patients with extensive prior cytotoxic exposure.

Third, this case demonstrates that aggressive supportive care can potentially prevent infectious complications during prolonged agranulocytosis (14). The patient survived three days with ANC 0 without infection. The institutional protocol included: (a) immediate transfer to laminar airflow unit (15); (b) broad-spectrum antibacterial and antifungal prophylaxis (16); and (c) combined long- and short-acting G-CSF plus IVIG (17). It should be noted that several of these interventions (e.g., combination G-CSF, IVIG) are not routinely recommended in current international guidelines for solid tumor patients, and represent institutional practice based on the perceived high risk in this individual case. The individual contribution of each intervention cannot be determined from a single case. Nevertheless, the overall strategy of early, intensive supportive care—initiated before fever or infection developed—appeared to be beneficial in this patient.

Fourth, this case suggests that invasive procedures may be feasible in selected neutropenic patients during early hematologic recovery. The successful thoracentesis at ANC 0.06×10^9^/L was performed under the following conditions: the patient was already on maximal antibiotic prophylaxis; the bone marrow had demonstrated the first sign of recovery; and the procedure was performed with strict asepsis for an urgent clinical need that could not be delayed. This single successful procedure does not support a general recommendation that thoracentesis can be safely performed during profound neutropenia. A more appropriate conclusion is that urgent invasive procedures may be feasible in carefully selected patients receiving intensive supportive care and after careful multidisciplinary assessment. The decision should always weigh the urgency of the procedure against the infectious and bleeding risks.

Published reports on chemotherapy-induced agranulocytosis have consistently demonstrated that the risk of severe neutropenia increases with cumulative cytotoxic exposure (18). Sacituzumab govitecan, which the patient received for 32 cycles, is associated with grade ≥3 neutropenia in approximately 50–60% of patients in clinical trials (19), further supporting the notion of compromised marrow reserve in this patient. Regarding cumulative bone marrow toxicity, pharmacokinetic/pharmacodynamic models have shown that bone marrow tolerance narrows with each sequential line of therapy, making it increasingly sensitive to subsequent genotoxic stress. Reports on hematologic toxicity following regional arterial infusion chemotherapy remain scarce; most published series have focused on hepatic arterial infusion and report predominantly mild-to-moderate myelosuppression (20). To our knowledge, no prior case has documented ANC reaching zero after reduced-dose pulmonary arterial infusion in a heavily pretreated TNBC patient. Our observation thus adds to the limited literature by illustrating the potential severity of myelotoxicity in this specific clinical context”.

The strengths of this case report include: This case report include complete clinical documentation and detailed supportive care timeline, which provide practical reference for clinicians. Published reports on chemotherapy-induced agranulocytosis emphasize that cumulative bone marrow toxicity increases with each line of therapy. Sacituzumab govitecan, which the patient received previously, is associated with significant neutropenia in clinical trials. Regional arterial infusion chemotherapy-related toxicity data are limited, but our case adds to the small body of evidence suggesting that even reduced-dose regional therapy can produce severe systemic toxicity in vulnerable patients.

The limitations are inherent to a single-case study: results are not generalizable; causality cannot be established; the specific mechanisms of myelosuppression were not explored; and long-term follow-up is needed.

## 4 Conclusion

This case illustrates that in heavily pre-treated patients, a pre-treatment ANC within the conventional safe range may not guarantee adequate marrow reserve, and even dose-reduced locoregional chemotherapy can potentially trigger life-threatening myelosuppression. The observation also highlights that proactive, intensive supportive care—initiated before infection develops—may be key to managing this high-risk situation. Furthermore, the successful thoracentesis in this patient suggests that necessary invasive procedures may be feasible in carefully selected patients during early hematologic recovery, under comprehensive risk-mitigation strategies and multidisciplinary evaluation. These observations are derived from a single case and should not be interpreted as modifying current clinical practice; rather, they underscore the need for individualized risk assessment and vigilant supportive care in this growing patient population.

## 5 Patient perspective

From the patient’s perspective, this period was one of extreme physical and emotional vulnerability. The relentless progression of her cancer had already caused severe dyspnea and discomfort. The sudden confinement to a laminar airflow unit, while essential for infection prevention, added a layer of isolation and psychological distress. She expressed profound gratitude for the intensive, round-the-clock care that brought her through the crisis without any signs of infection, which she feared would be her “final battle.” The successful management of her acute dyspnea via thoracentesis, despite the risks, provided immense relief and restored a degree of hope and the ability to communicate easily with her family for a time during her advanced illness.

## Data Availability

The original contributions presented in the study are included in the article/**Supplementary Material**. Further inquiries can be directed to the corresponding author.
